# Relationship between Depression Symptoms and Different Types of Measures of Obesity (BMI, SAD) in US Women

**DOI:** 10.1155/2020/9624106

**Published:** 2020-11-22

**Authors:** Yang Zhou, Guifang Yang, Wen Peng, Hongliang Zhang, Zhenyu Peng, Ning Ding, Tao Guo, Yuzhong Cai, Qijian Deng, Xiangping Chai

**Affiliations:** ^1^Department of Emergency Medicine, Second Xiangya Hospital, Central South University, Changsha, China; ^2^Emergency Medicine and Difficult Diseases Institute, Second Xiangya Hospital, Central South University, Changsha, China; ^3^Department of Psychiatry, Second Xiangya Hospital, Central South University, Changsha, China

## Abstract

**Objective:**

To estimate the relationship between obesity (defined by both BMI and SAD) and various levels of depressive symptoms in women in the United States.

**Methods:**

This is a cross-sectional design. All data were collected from NHANES 2011-2012 and 2013-2014. The Patient Health Questionnaire (PHQ-9) was the primary variable used to index depressive symptoms. SAD was assessed using an abdominal caliper. We stratified participates into three groups according to SAD (trisection): T1: low (11.8-18.4 cm), T2: middle (18.5-22.8 cm), and T3: high (22.9-40.1 cm). Other data were collected following the NHANES protocols. We aimed to investigate the effects of obesity on the depression in the NHANES populations.

**Results:**

A total of 4477 women were enrolled in the final study population. Participants with a high SAD had the highest risk of clinical depression symptoms (OR = 1.2, 95% CI: 1.1-1.4), which was, in particular, the case for moderate-severe depression (OR = 1.4, 95% CI: 1.1-1.7) and severe depression (OR = 1.4, 95% CI: 1.0-1.9). We also found a significant relationship between SAD and BMI (*r* = 0.836). We did, however, not find a significant relationship between BMI and severe depression.

**Conclusions:**

SAD had a better correlation with clinical depression symptoms than BMI, especially regarding severe depression symptoms.

## 1. Introduction

Depression is a serious mental disorder with emotional and physical behavioral symptoms [1, 2]. The World Health Organization reported that depression currently affects more than 300 million people. The National Alliance on Mental Illness (NAMI) reported that 7.2% (17.7 million people) of people experience a major depressive episode in the United States. Depression has become a massive global health problem and costs the economy trillions of dollars worldwide [3]. Women are more likely to be affected by depression and are twice as much at risk for a history of depressive episodes in their lifetime than men [4–7]. Women with anxiety have a higher burden of associated illnesses, including work absenteeism and comorbid major depressive disorder [8]. Evidence indicates that a sustained depressive mood is a gateway symptom for a major depressive disorder [9]. There is increasing interest to understand the health risks of clinical depressive symptoms due to the high prevalence coupled with their predictive validity as precursors for clinically diagnosed depression in women.

Obesity is a risk factor that could assist in identifying women who may require further assessment for risk factors for depression. A contributing factor to obesity may be depression that affects obesity-related behaviors such as lifestyle and psychological factors [9, 10]. Physical activity attenuated the relationship between depression and body composition change for young women [11]. A systematic review of 34 studies found that 75% of patients preferred psychotherapy over drug treatment, although severe depression patients were more preferring drugs [12]. In a cross-sectional study in US, young women and Hispanics were more likely to develop into depressive symptoms than nonobese women [9]. Similarly, a large cohort study suggested that obesity, female gender, and low education may serve as targets for early detection, prevention, and intervention in this population [13]. Moreover, female gender, low education, and extreme obesity were associated with severe depression [14].

Although some of the previous studies have reported the risk factors of depressive symptoms in women, studies have quantified the strong reciprocal association between risk factors for depression and obesity. Recent findings suggest that the sagittal abdominal diameter (SAD), also known as “abdominal height,” can be used as a noninvasive method to index visceral fat [15–20]. Moreover, visceral fat has a greater association with a myriad of metabolic disturbances than overall obesity. Studies indicate that the SAD is better than the BMI in recognizing cardiovascular risk factors [19], chronic kidney disease [21], cardiometabolic disorders [17], and glucose metabolism [16]. Increased fasting blood glucose, total cholesterol (TC), triglyceride (TG), and low-density lipoprotein cholesterol (LDL) and decreased high-density lipoprotein cholesterol (HDL) are associated with depression [22–24]. Besides, several large cohort studies have shown the prognostic value of SAD in general and heart disease populations [21]. However, thus far, no studies have involved different anthropometric approaches related to various depression symptoms.

Therefore, in the current study, we investigated the association between obesity (defined by both BMI and SAD) and different levels of depressive symptoms in women.

## 2. Methods

### 2.1. Participants and Study Design

**Figure 1:**
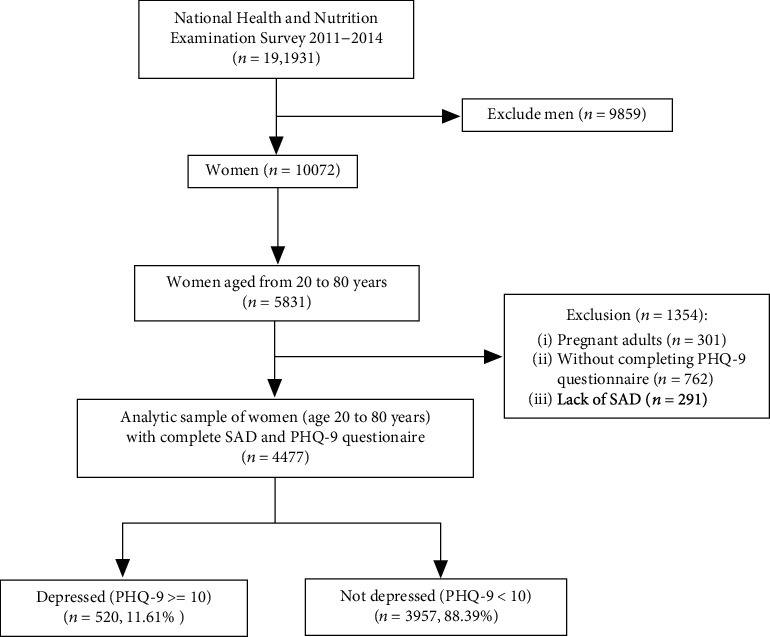
Flow chart of the participants' enrollment.

The National Health and Nutrition Examination Survey (NHANES) is a cross-sectional survey of the population of the USA. The survey is unique in that it combines interviews and physical examinations. We analyzed data derived from the NHANES obtained between 2011-2012 and 2013-2014. The NHANES protocol had been approved by the US National Center for Health Statistics Research Ethics Review Board, and all participants provided informed consent. From a total sample of 19931 participants who were interviewed for NHANES between 2011 and 2014, a subsample of 4477 participants was eligible for our study. The male sex participants, aged under 20 years old, pregnant women, those without complete sagittal abdominal diameter (SAD) measurements, and the Patient Health Questionnaire (PHQ-9) was answered with “refused” and “do not know” were excluded (Figure 1). Further background on NHANES can be acquired at https://www.cdc.gov/nchs/nhanes/default.aspx.

### 2.2. Anthropometry

#### 2.2.1. Sagittal Abdominal Diameter (SAD)

SAD was measured by an abdominal caliper (Holtain Kahn Abdominal Caliper) in the supine position with the hips in a flexed, relaxed position, and the examiner marked the iliac crests' midpoint [15, 16]. Then, the lower arm of the caliper was placed under the back, and the upper arm was raised above the abdomen to align with the top. The SAD value was the distance between the back of the iliac crest and the front of the abdomen. Two measurements were taken for SAD, and the average was used to determine the SAD value. If the difference between the first and second AD measurements was greater than 0.5 cm, the three closest SAD readings were used to obtain the average SAD value. All four readings were used to get the mean value of the SAD in cases where the two outlying measurements are equal to the two closest measurements [25]. In our study, we defined the SAD of each participant as the average of two initial measurements or up to four measurements, as specified in the NHANES online analysis instructions [15]. According to the SAD of individuals at the baseline, three groups (trisection) were categorized as T1: low (11.8-18.4 cm), T2: middle (18.5-22.8 cm), and T3: high (22.9-40.1 cm).

#### 2.2.2. Body Mass Index (BMI)

The BMI was calculated as weight in kilograms divided by height in meters squared and then rounded to one decimal. The cut-off criteria used were based on the US Centers for Disease Control and Prevention's sex-specific 2000 BMI-for-age growth charts.

#### 2.2.3. Study Variables

We chose covariates as potential confounding factors based on previous research. Our association analysis included the following covariates: age, race, body mass index (BMI), education level, family income-to-poverty ratio (PIR), marital status, diabetes mellitus, smoking status, alcohol consumption, health insurance, hypertension, hypercholesterolemia, triglyceride (TG), total cholesterol (TC), high-density lipoprotein cholesterol (HDL-c), low-density lipoprotein cholesterol (LDL-c), and fasting blood glucose. Race was classified as non-Hispanic white, non-Hispanic black, Mexican-American, other Hispanic, or other race. We categorized education level based on classified college graduate or above, high school graduate, or less than 9^th^ grade. Marital status was divided into living with a partner, separated, married, never married, divorced, or widowed. The variable Family PIR was calculated by dividing the household income by the poverty guidelines (specific to family size) and the corresponding year and state, which has been used in previous studies. The definition of smoking status and alcohol consumption was based on previous reports. The diagnosis of hypertension was made when the participants had been told by their doctor that they had high blood pressure or if they were taking antihypertensive drugs. The diagnosis of hypercholesterolemia was based on the participant's report, if told by a doctor that they had high cholesterol or used lipid-lowering drugs. The variable health insurance was acquired from the response to a question: “Do you have health insurance or other kinds of health insurance?” The diagnosis of diabetes mellitus was made if participants reported being told by their doctor that they had diabetes or sugar diabetes.TC, TG, HDL-c, LDL-c, and fasting blood glucose were measured as described in the NHANES Laboratory Procedures Manual.

#### 2.2.4. Outcome Data

Depressive symptoms were assessed using the PHQ-9, an effective 9-item depression screen, which questions the frequency of depression symptoms in the past two weeks. The Patient Health Questionnaire (PHQ-9) had been provided a consistent, evidence-based approach for calculating PHQ-9 subscale scores by a cross-section study of NHNAES (2005-2016) [26]. Each item was scored on a scale of 0-3, with a total score ranging from 0 to 27. Based on these scores, depressive symptoms could be divided into “none or minimum” (0-4), “mild” (5-9), “moderate” (10-14), “moderately severe” (15-19), and “severe” (20-27). For prior analyses, participants who scored ≥10 or more were indicated as having clinically relevant depression [1].

#### 2.2.5. Statistical Analysis

For baseline characteristics of the participants, we used mean ± standarddeviation or interquartile ranges (IQRs) for continuous variables depending on the value distribution. Categorical variables were presented as percentage or frequency and were assessed using chi-square analysis. Multivariate regression models were used to test the relationship between SAD (per 5 cm) and depression symptoms of various levels after adjustment of other variables. To further examine whether SAD was correlated with symptoms of clinical depression, three models were established based on the level of depression symptoms, as defined earlier. To evaluate the adjusted depression symptoms and SAD, the T1 (low group of SAD) was used as a reference; three models were constructed after adjustment for age, race, marital status, education level, smoking status, diabetes mellitus, alcohol consumption, hypertension, hyperlipemia, health insurance, family PIR, and fasting blood glucose. A smoothing spline curve technique was used to study the shape of the relationship between the SAD and BMI and various levels of depression symptoms adjusted for the abovementioned confounding factors. The association between SAD and BMI was tested by Pearson's correlation coefficient. Subgroup analysis and interaction were performed basing on all variables of Table 1. These analyses were carried out using R (https://www.R-project.org) and Empower (http://www.empowerstats.com, X&Y Solutions Inc., Boston, MA). Two-sided *p* values < 0.05 were considered statistically significant.

## 3. Results

### 3.1. Baseline Characteristics

After exclusion, a total of 4477 individuals from the NHANES (2011-2014) were enrolled in this study (Figure 1). The mean age was 49.5 ± 17.2 years; 11.61% (*n* = 520) participants had the depressive symptoms based on PHQ-9. According to the SAD, the participants were divided into three groups (trisection).  Table 1 shows the baseline demographic characteristics, laboratory examination, and medical history. The high SAD group participants were older, more likely to be non-Hispanic black, widowed, divorced, and separated and had a high proportion of current smoking, former smoking, diabetes, hypertension, hyperlipemia, BMI, TC, and TG was comparable to other groups, while the family PIR, HDL-c, and health insurance were less common than in the T1 and T2 groups (Table 1).

### 3.2. Symptoms of Depression

Out of the total population studied, 520 women had clinically relevant depression symptoms in the past two weeks, of whom 311 (59.8%) had moderate depression symptoms, 141 (27.1%) had moderate-severe depression symptoms, and 68 (13.1%) had severe depression symptoms. The different levels of symptoms of depression among the SAD categories are displayed in Table 2. T3 group participants were more likely to have depression than participants in T1 and T2 (Table 2).

### 3.3. Multivariate Adjusted Analyses

**Figure 2:**
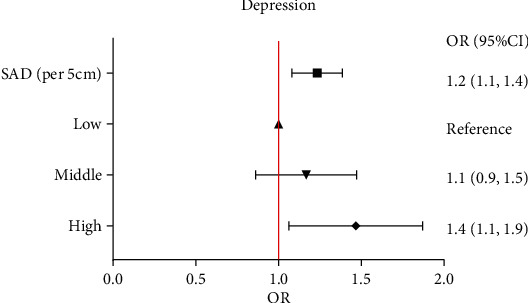
Depressive symptoms by the SAD category. All models are adjusted for age, race, marital status, education level, smoking status, diabetes mellitus, alcohol consumer, hypertension, hyperlipemia, health insurance, family PIR, and fasting blood glucose.

After adjustment for clinically relevant confounders and covariates, we found that every 5 cm increase in SAD leads to an increase of 20% of depression symptoms, for which the adjusted OR was 1.2 (95% CI: 1.1-1.4, *p* < 0.01) (Table 3). We also converted the SAD from a continuous variable to a categorical variable (trisection); the group participants with the highest SAD had the highest risk of depression symptoms, with the adjusted OR of 1.4 (95% CI: 1.1-1.9, *p* = 0.02) as compared to the low SAD reference group (Figure 2(a)). A similar pattern was observed for participants with severe depression. The highest risk of severe depression was found in the high SAD group, followed by the middle SAD group, whereas the lowest severe depression symptoms rate was found in the low SAD group (Figure 2(d)). The adjusted ORs were 2.5 (95% CI: 1.1-5.9, *p* = 0.03) for the high SAD group and 1.6 (95% CI: 0.7-3.9, *p* = 0.30) for the middle SAD group, with low SAD group participants as the reference group. No significant difference was noted between groups concerning the incidences of moderate depression and moderately severe depression (Figures 2(b) and 2(c)).

### 3.4. The Linear-Shaped Relationship between SAD and Depression Symptoms

**Figure 3:**
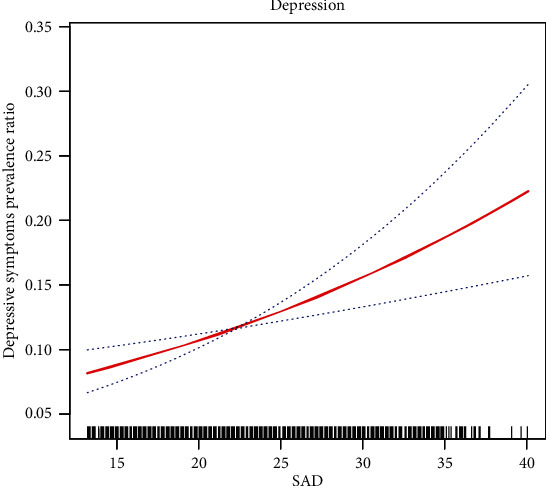
Smooth spline curves of SAD for the estimation of risk of depressive symptoms after adjusting multivariate rates. Red lines denote fitted curves and blue lines represent 95% confidence intervals for the association between SAD and depressive symptoms. All models are adjusted for the confounders in Figure 2.

We used the SAD as a continuous variable to study its relationship with depression and different levels of symptoms of clinical depression after adjustment in a multivariate analysis. We found a nearly linear relationship with depression and with various levels of symptoms of clinical depression (Figure 3).

### 3.5. The Linear-Shaped Relationship between BMI and Depression Symptoms

**Figure 4:**
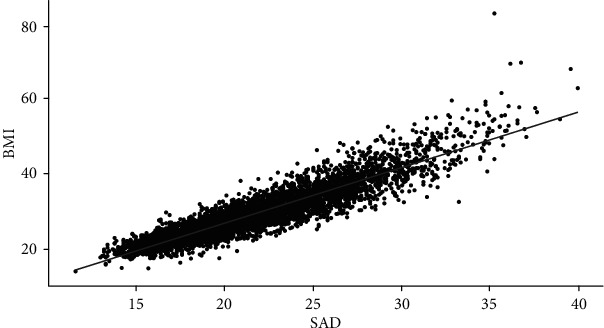
Correlation and agreement between SAD and body mass index (BMI).

**Figure 5:**
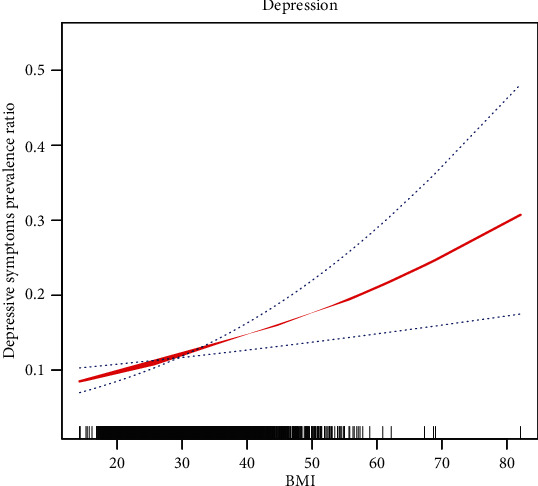
Smooth spline curves of BMI for the estimation of risk of depressive symptoms after adjusting multivariate rates. Red lines denote fitted curves, and blue lines represent 95% confidence intervals for the association between BMI and depressive symptoms. All models are adjusted for the confounders in Figure 2.

We observed a significant correlation between SAD and BMI (*r* = 0.836, *p* < 0.05; Figure 4). We used BMI as a continuous variable to study the relationship between BMI and depression and different levels of clinical depression symptoms after adjustment for confounding variables and found correlations similar to those between the SAD and the depression groups (Figure 5, Table S1).

### 3.6. Subgroup Analysis

We used age, marital status, education level, family PIR, health insurance, diabetes mellitus, smoking status, alcohol consumption, hypercholesterolemia, and hypertension as the stratification variables to observe the trend of effect sizes in these variables (Figure S1, S2, S3, and S4).

## 4. Discussion

To our best knowledge, this is the first investigation to assess the SAD among women with symptoms of depression. We found that depression symptoms are associated with the SAD. Specifically, the primary results of our study could be summarized as follows: (1) moderate, moderately severe, and severe depression symptoms are more frequently observed in a people with a high level of SAD; (2) SAD is correlated with BMI, and SAD is a better predictor of severe depression symptoms than BMI; and (3) interaction and subgroup analyses suggested that the associations of SAD and different levels of depression symptoms were stable. We did not find any statistically significant differences between groups in terms of moderate and moderately severe depression symptoms. We found that a high SAD level is positively associated with severe depression symptoms even after adjusting for other covariates.

Depression and obesity are both risk factors of adverse health outcomes. Previous studies have compared the use of various obesity measurements in populations to predict depression symptoms. Staiano et al. measure subcutaneous and visceral adiposity using dual-energy X-ray and tested the associations between these variables and depressive symptoms among 59 nonobese adults over a two-year period [11]. Further, they found that BMI, fat mass, subcutaneous adiposity, and increased weight are related to depression for young women. Vogelzangs et al. conducted a longitudinal study spanning five years with 2088 participants aged 70–79 years and found that baseline depression was associated with SAD and visceral fat [27]. Everson-Rose et al. conducted a cross-sectional study examining association between depressive symptoms and visceral adipose tissue measured by CT in a sample of 409 middle-aged women [28]. This study showed that the relationship between depressive symptoms and visceral adipose tissue was strongest in obese and overweight women. Our study confirmed previous investigations of the association between SAD and BMI increased the depressive symptoms.

SAD is a potential health concern and is regarded as an index for visceral fat with an increased risk of cardiometabolic disorders and glucose metabolism [16, 17]. The strong associations between these factors may be explained by visceral obesity, which indicates an organic etiology of depression symptoms. SAD is a noninvasive method to index visceral fat [15–20], which releases higher concentrations of adipokines that are associated with proinflammatory processes [29]. Furthermore, inflammation is known to play an important role in depression, providing one mechanism underlying this relationship [30]. Other authors suggested that self-identification as ‘overweight' is significantly associated with depression symptoms [31]. Weight status was related with the depression [32, 33]. Therefore, the relationship between a high level of SAD and severe depression symptoms among women may be explained by the notion that women are more likely to pay attention to the SAD and weight loss [34].

We also found that the predictive ability of the SAD measurement is particularly noteworthy compared to BMI measurements when subjects were stratified by depression symptoms. Additionally, we observed a high BMI appeared to have little significance for predicting severe depression symptoms, while SAD's improved predictive value was apparent among participants with severe depression symptoms. Our results support an association between obesity and depression but demonstrate that depressive symptoms were more related to abdominal obesity not to body mass [35]. It is consistent with the hypothesis that obese individuals may develop depression due to the adverse effects of the proinflammatory state of excess adiposity on the central nervous system. Although we cannot determine which, if any, of these models explain the association between depressive symptoms and obesity, this study shows that depressive symptoms result in an increase in abdominal obesity, more than overall obesity, suggesting that there may be specific pathophysiological mechanisms which link depression with visceral fat accumulation.

The strength of the present study was its large sample size representative of the US adult population. Moreover, the ability to predict SAD measurements was particularly noteworthy compared to BMI measurements when subjects were stratified by different depression symptoms and these methods are costly and not feasible in clinical practice. Furthermore, the SAD measurement is entirely focused on abdominal height, which better reflects visceral adiposity.

The present study also had some weaknesses. Due to its cross-sectional design, the causality of the relationships between the anthropometric measurements and depression could not be studied in more detail. Moreover, we were unable to collect some factors, such as the psychological, behavior, and lifestyle. A further limitation is the evaluation of visceral fat only through SAD, since computerized tomography identifying the percentage of visceral adipose tissue would be more desirable. However, previous studies have reported that SAD is an alternative indicator of abdominal adipose tissue enlargement [16, 17, 36, 37].

## 5. Conclusions

SAD had a better correlation with clinical depression symptoms than BMI, especially regarding severe depression symptoms. This investigation has laid the foundation for further research on the ability of various anthropometric measurements (SAD and BMI) to distinguish individuals with different symptoms of depression.

## Figures and Tables

**Table 1:** Baseline demographic, history disease, and laboratory examination in US adults of women aged from 20 to 80 years in NHANES 2011-2014.

Characteristics	Total (*n* = 4477)	SAD (cm)			*p* value
		T1 (11.8-19.7) (*n* = 1476)	T2 (19.8-23.9) (*n* = 1484)	T3 (24.0-40.1) (*n* = 1517)	
Demographics					
Age (year, mean ± SD)	49.5 ± 17.2	44.3 ± 17.6	51.8 ± 17.0	52.3 ± 15.7	<0.01
BMI (kg/m^2^, mean ± SD)	29.3 ± 7.3	22.7 ± 2.7	28.2 ± 3.2	36.9 ± 6.2	<0.01
Race (*n*, %)					<0.01
Mexican American	503 (11.2)	127 (8.6)	190 (12.8)	186 (12.3)	
Other Hispanic	460 (10.3)	154 (10.4)	171 (11.5)	135 (8.9)	
Non-Hispanic white	1844 (41.2)	640 (43.4)	604 (40.7)	600 (39.6)	
Non-Hispanic black	1027 (22.9)	164 (11.1)	346 (23.3)	517 (34.1)	
Non-Hispanic Asian	519 (11.6)	339 (23.0)	135 (9.1)	45 (3.0)	
Other race	124 (2.8)	52 (3.5)	38 (2.6)	34 (2.2)	
Marital status (*n*, %)					<0.01
Married	2075 (46.4)	713 (48.3)	714 (48.2)	648 (42.7)	
Widowed	465 (10.4)	103 (7.0)	178 (12.0)	184 (12.1)	
Divorced	607 (13.6)	148 (10.0)	207 (14.0)	252 (16.6)	
Separated	176 (3.9)	52 (3.5)	47 (3.2)	77 (5.1)	
Never married	852 (19.0)	341 (23.1)	242 (16.3)	269 (17.7)	
Living with partners	299 (6.7)	119 (8.1)	94 (6.3)	86 (5.7)	
Education level (*n*, %)					<0.01
Less than 9th grade	323 (7.2)	84 (5.7)	116 (7.8)	123 (8.1)	
High school graduate	1490 (33.3)	375 (25.4)	504 (34.0)	611 (40.3)	
College graduate/above	2664 (59.5)	1017 (68.9)	864 (58.2)	783 (51.6)	
Smoking status (*n*, %)					<0.01
Never smoking	2910 (65.1)	1050 (71.2)	975 (65.7)	885 (58.5)	
Current smoking	759 (17.0)	210 (14.2)	251 (16.9)	298 (19.7)	
Former smoking	803 (18.0)	215 (14.6)	258 (17.4)	330 (21.8)	
Diabetes mellitus (*n*, %)	427 (10.0)	90 (6.4)	117 (8.2)	220 (15.1)	<0.01
Alcohol consumer (*n*, %)	2791 (62.4)	983 (66.8)	898 (60.5)	910 (60.1)	<0.01
Hypertension (*n*, %)	1709 (38.2)	300 (20.4)	569 (38.4)	840 (55.4)	<0.01
Hyperlipemia (*n*, %)	1656 (37.2)	379 (25.8)	593 (40.1)	684 (45.4)	<0.01
Health insurance (*n*, %)	3587 (80.2)	1190 (80.7)	1190 (80.3)	1207 (79.7)	0.80
Family PIR	2.3 ± 1.6	2.6 ± 1.7	2.3 ± 1.6	1.9 ± 1.4	<0.01
TC (mmol/L, mean ± SD)	5.1 ± 1.1	4.9 ± 1.0	5.1 ± 1.1	5.1 ± 1.1	<0.01
TG (mmol/L, IQR)	1.1 (0.7-1.5)	0.8 (0.6-1.2)	1.1 (0.8-1.6)	1.3 (0.9-1.8)	<0.01
HDL-c (mmol/L, mean ± SD)	1.5 ± 0.4	1.7 ± 0.4	1.5 ± 0.4	1.3 ± 0.3	<0.01
LDL-c (mmol/L,mean ± SD)	2.9 ± 0.9	2.8 ± 0.9	3.1 ± 0.9	3.0 ± 0.8	<0.01
Fasting blood glucose (mmol/L, mean ± SD)	5.9 ± 1.8	5.7 ± 1.3	5.9 ± 1.8	5.9 ± 2.1	0.28

Note: results weighted to represent the United States. (1) A ratio of family income to poverty <1 indicates a family that is living in poverty. (2) NHANES participants over 80 y of age are top-coded at 80 y of age. Abbreviations: SAD: sagittal abdominal diameter; BMI: body mass index; Family PIR: a ratio of family income to poverty; TG: triglyceride; TC: total cholesterol; HDL-c: high-density lipoprotein cholesterol; LDL-c: low-density lipoprotein cholesterol; IQR: interquartile ranges.

**Table 2:** The different levels of depressive symptoms among US women.

	SAD (cm)			*p* value
	T1 (11.8-19.7)	T2 (19.8-23.9)	T3 (24.0-40.1)	
Depression, *n* (%)	117 (7.9%)	161 (10.8%)	242 (16.0%)	<0.01
Moderate depression, *n* (%)	76 (5.1%)	103 (6.9%)	132 (8.7%)	<0.01
Moderately severe, *n* (%)	28 (1.9%)	40 (2.7%)	73 (4.8%)	<0.01
Severe, *n* (%)	13 (0.9%)	18 (1.2%)	37 (2.4%)	<0.01

Abbreviations: SAD: sagittal abdominal diameter.

**Table 3:** Multivariable linear regression analyzed the association of SAD (per 5 cm) and symptoms of depression in US adult women aged from 20 to 80 years in NHANES 2011-2014.

	OR (95% CI), *p* value		
	Model 1	Model 2	Model 3
Depression			
SAD (per 5 cm)	1.5 (1.3, 1.6), <0.01	1.4 (1.3, 1.6), <0.01	1.2 (1.1, 1.4), <0.01
Low (11.8-19.7 cm)	Ref	Ref	Ref
Middle (19.8-23.9 cm)	1.4 (1.1, 1.8), <0.01	1.3 (1.0, 1.7), 0.03	1.1 (0.9, 1.5), 0.35
High (24.0-40.1 cm)	2.2 (1.7, 2.8), <0.01	2.1 (1.6, 2.7), <0.01	1.4 (1.1, 1.9), 0.02
Moderate depression			
SAD (per 5 cm)	1.3 (1.2, 1.5), <0.01	1.3 (1.1, 1.4), <0.01	1.1 (0.9, 1.3), 0.24
Low (11.8-19.7 cm)	Ref	Ref	Ref
Middle (19.8-23.9 cm)	1.4 (1.0, 1.9), 0.04	1.3 (0.9, 1.7), 0.13	1.0 (0.7, 1.4), 0.85
High (24.0-40.1 cm)	1.8 (1.3, 2.4), <0.01	1.6 (1.2, 2.2), <0.01	1.1 (0.8, 1.6), 0.60
Moderately severe depression			
SAD (per 5 cm)	1.7 (1.4, 2.0), <0.01	1.7 (1.4, 2.0), <0.01	1.4 (1.1, 1.7), <0.01
Low (11.8-19.7 cm)	Ref	Ref	Ref
Middle (19.8-23.9 cm)	1.4 (0.9, 2.3), 0.15	1.5 (0.9, 2.4), 0.14	1.4 (0.8, 2.5), 0.19
High (24.0-40.1 cm)	2.6 (1.7, 4.1), <0.01	2.6 (1.7, 4.2), <0.01	1.6 (0.9, 2.8), 0.06
Severe depression			
SAD (per 5 cm)	1.5 (1.2, 1.9), <0.01	1.5 (1.1, 1.9), <0.01	1.4 (1.0, 1.9), 0.04
Low (11.8-19.7 cm)	Ref	Ref	Ref
Middle (19.8-23.9 cm)	1.4 (0.7, 2.8), 0.38	1.3 (0.6, 2.6), 0.53	1.6 (0.7, 3.9), 0.30
High (24.0-40.1 cm)	2.8 (1.5, 5.3), <0.01	2.6 (1.3, 5.1), <0.01	2.5 (1.1, 5.9), 0.03

Model 1: crude model. Model 2: adjusted for age, and race. Model 3: adjusted for age, race, marital status, education level, smoking status, diabetes mellitus, alcohol consumer, hypertension, hyperlipemia, health insurance, family PIR, and fasting blood glucose. Abbreviations: SAD:L sagittal abdominal diameter; OR: odds ratio; CI: confidence interval.

## Data Availability

The NHANES data of this study are openly available at https://www.cdc.gov/nchs/nhanes/default.aspx.

## Supplementary Materials

Supplementary 1 Tables S1: multivariable linear regression analyzed the association of BMI and symptoms of depression in US adult women aged from 20 to 80 years in NHANES 2011-2014.

Supplementary 2 Figure S1: forest plot showing the odds ratios, confidence intervals, and *p* value for the interaction of SAD and depression in the different groups. Figure S2: forest plot showing the odds ratios, confidence intervals, and *p* value for the interaction of SAD and moderate depression in the different groups. Figure S3: forest plot showing the odds ratios, confidence intervals, and *p* value for the interaction of SAD and moderate severe depression in the different groups. Figure S4: forest plot showing the odds ratios, confidence intervals, and *p* value for the interaction of SAD and severe depression in the different groups.
