# Supplementary material for: Relationship between Depression Symptoms and Different Types of Measures of Obesity (BMI, SAD) in US Women
Source: Behav Neurol. 2020 Nov 22;2020:9624106. doi: 10.1155/2020/9624106 (PMC7705436; doi:10.1155/2020/9624106)
Supplement: Supplementary 1 — Tables S1: multivariable linear regression analyzed the association of BMI and symptoms of depression in US adult women aged from 20 to 80 years in NHANES 2011-2014. [file 9624106.f1.doc]

**Tables S1.** Multivariable linear regression analyzed the association of BMI and symptoms of depression in US adult women aged from 20 to 80 years in NHANES 2011-2014

|  |  | OR (95%CI), *P*-value |  |
| --- | --- | --- | --- |
|  | Model 1 | Model 2 | Model 3 |
| Depression | 1.04 (1.03, 1.05) <0.001 | 1.04 (1.03, 1.05) <0.001 | 1.02 (1.01, 1.04) 0.002 |
| Moderate depression | 1.03 (1.01, 1.04) <0.001 | 1.02 (1.01, 1.04) 0.003 | 1.01 (0.99, 1.03) 0.276 |
| Moderately severe depression | 1.06 (1.04, 1.08) <0.001 | 1.06 (1.03, 1.08) <0.001 | 1.04 (1.01, 1.06) 0.002 |
| Severe depression | 1.03 (1.00, 1.06) 0.036 | 1.03 (1.00, 1.06) 0.087 | 1.02(0.97, 1.06) 0.228 |

Model 1: crude model

Model 2: adjusted for age, and race;

Model 3: adjusted for age, race, marital status, education level, smoking status, diabetes mellitus, alcohol consumer, hypertension, hyperlipemia, health insurance, family PIR, and fasting blood glucose.

Abbreviations: BMI, body mass index; OR, odds ratio; CI, confidence interval.
