# Supplementary material for: Relationship between Depression Symptoms and Different Types of Measures of Obesity (BMI, SAD) in US Women
Source: Behav Neurol. 2020 Nov 22;2020:9624106. doi: 10.1155/2020/9624106 (PMC7705436; doi:10.1155/2020/9624106)

Figure legends

Figure S1. Forest plot showing the odds ratios, confidence intervals, and P-value for interaction of SAD and depression in the different groups.

Figure S2. Forest plot showing the odds ratios, confidence intervals, and P-value for interaction of SAD and moderate depression in the different groups.

Figure S3. Forest plot showing the odds ratios, confidence intervals, and P-value for interaction of SAD and moderate severe depression in the different groups.

Figure S4. Forest plot showing the odds ratios, confidence intervals, and P-value for interaction of SAD and severe depression in the different groups.

(S1)

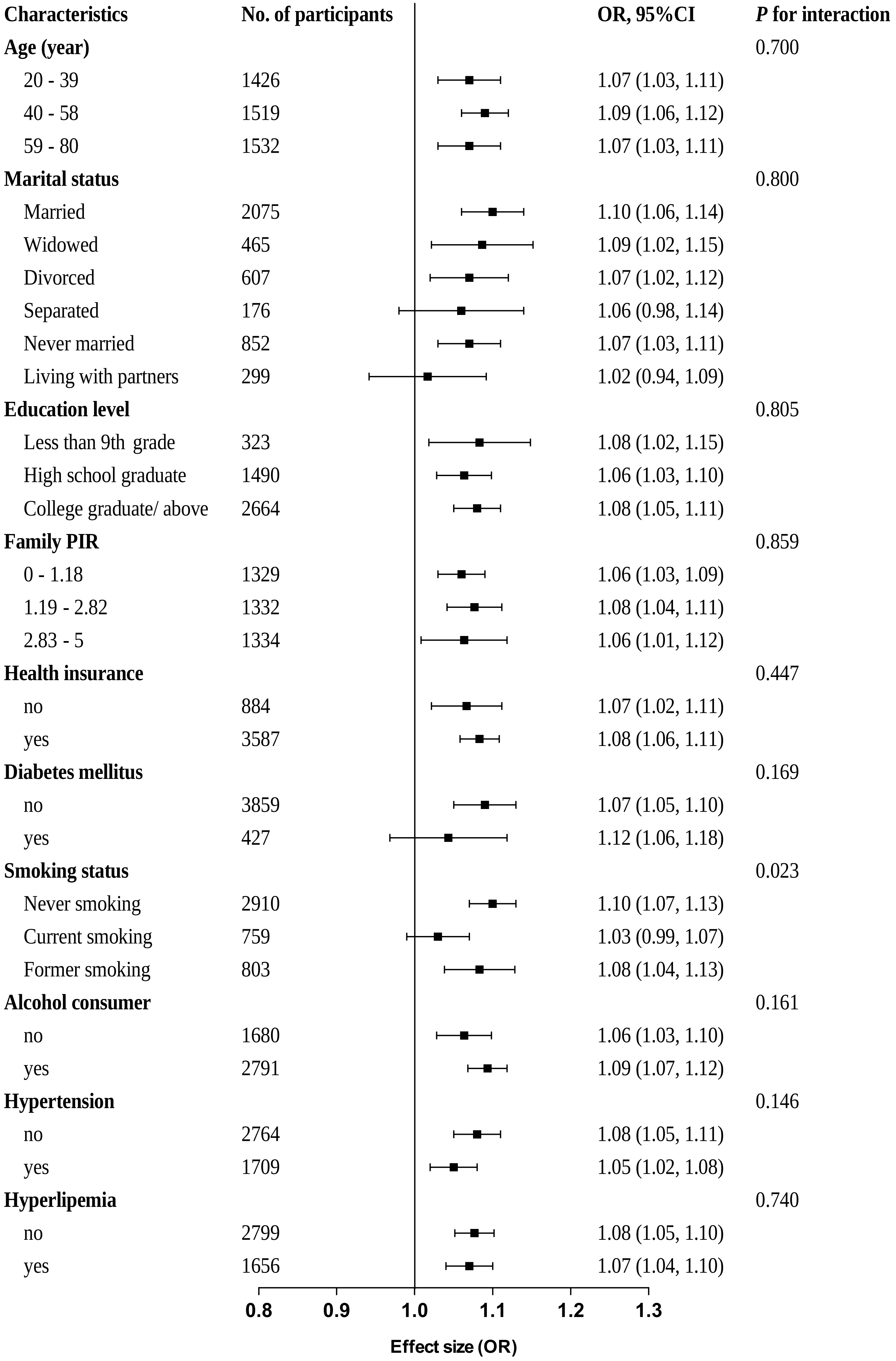

Characteristics
No. of participants
OR, 95%CI
P for interaction
Age (year)
0.700
20 - 39
1426
1.07 (1.03, 1.11)
40 - 58
1519
1.09 (1.06, 1.12)
59 - 80
1532
1.07 (1.03, 1.11)
Marital status
0.800
Married
2075
1.10 (1.06, 1.14)
Widowed
465
1.09 (1.02, 1.15)
Divorced
607
1.07 (1.02, 1.12)
Separated
176
1.06 (0.98, 1.14)
Never married
852
1.07 (1.03, 1.11)
Living with partners
299
1.02 (0.94, 1.09)
Education level
0.805
Less than 9th grade
323
1.08 (1.02, 1.15)
High school graduate
1490
1.06 (1.03, 1.10)
College graduate/ above
2664
1.08 (1.05, 1.11)
Family PIR
0.859
0 - 1.18
1329
1.06 (1.03, 1.09)
1.19 - 2.82
1332
1.08 (1.04, 1.11)
2.83 - 5
1334
1.06 (1.01, 1.12)
Health insurance
0.447
no
884
1.07 (1.02, 1.11)
yes
3587
1.08 (1.06, 1.11)
Diabetes mellitus
0.169
no
3859
1.07 (1.05, 1.10)
yes
427
1.12 (1.06, 1.18)
Smoking status
0.023
Never smoking
2910
1.10 (1.07, 1.13)
Current smoking
759
1.03 (0.99, 1.07)
Former smoking
803
1.08 (1.04, 1.13)
Alcohol consumer
0.161
no
1680
1.06 (1.03, 1.10)
yes
2791
1.09 (1.07, 1.12)
Hypertension
0.146
no
2764
1.08 (1.05, 1.11)
yes
1709
1.05 (1.02, 1.08)
Hyperlipemia
0.740
no
2799
1.08 (1.05, 1.10)
yes
1656
1.07 (1.04, 1.10)
0.8
0.9
1.0
1.1
1.2
1.3
Effect size (OR)

(S2)

Characteristics	No. of participants	OR, 95%CI	*P* for interaction
**Age (year)**			0.534
20 - 39	1426	1.03 (0.98, 1.08)	
40 - 58	1519	1.06 (1.02, 1.10)	
59 - 80	1532	1.06 (1.02, 1.12)	
**Marital status**			--
Married	2075	1.08 (1.03, 1.13)	
Widowed	465	1.10 (1.02, 1.18)	
Divorced	607	1.03 (0.97, 1.09)	
Separated	176	1.08 (0.98, 1.19)	
Never married	852	1.03 (0.98, 1.08)	
Living with partners	299	0.97 (0.88, 1.06)	
**Education level**			0.626
Less than 9th grade	323	1.07 (0.99, 1.16)	
High school graduate	1490	1.03 (0.99, 1.08)	
College graduate/ above	2664	1.06 (1.02, 1.09)	
**Family PIR**			0.784
0 - 1.18	1329	1.04 (1.01, 1.08)	
1.19 - 2.82	1332	1.03 (0.99, 1.08)	
2.83 - 5	1334	1.06 (1.00, 1.12)	
**Health insurance**			0.107
no	884	1.01 (0.96, 1.07)	
yes	3587	1.07 (1.04, 1.10)	
**Diabetes mellitus**			0.749
no	3859	1.06 (1.03, 1.09)	
yes	427	1.04 (0.97, 1.12)	
**Smoking status**			0.004
Never smoking	2910	1.09 (1.05, 1.13)	
Current smoking	759	0.99 (0.95, 1.03)	
Former smoking	803	1.05 (0.99, 1.11)	
**Alcohol consumer**			0.283
no	1680	1.04 (0.99, 1.08)	
yes	2791	1.07 (1.04, 1.10)	
**Hypertension**			0.607
no	2764	1.05 (1.01, 1.09)	
yes	1709	1.03 (1.00, 1.07)	
**Hyperlipemia**			0.727
no	2799	1.04 (1.01, 1.08)	
yes	1656	1.05 (1.01, 1.09)	

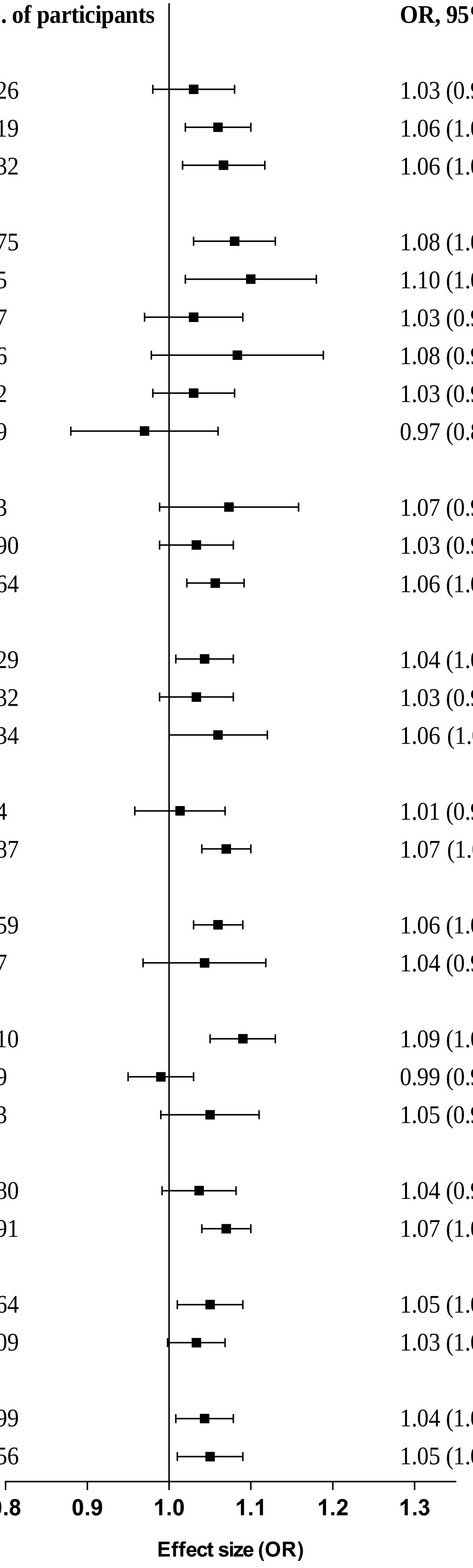

(S3)

Characteristics	No. of participants	OR, 95%CI	*P* for interaction
**Age (year)**			0.552
20 - 39	1426	1.11 (1.05, 1.18)	
40 - 58	1519	1.13 (1.07, 1.19)	
59 - 80	1532	1.07 (1.00, 1.15)	
**Marital status**			--
Married	2075	1.13 (1.07, 1.20)	
Widowed	465	1.07 (0.95, 1.20)	
Divorced	607	1.14 (1.05, 1.24)	
Separated	176	0.96 (0.84, 1.11)	
Never married	852	1.12 (1.05, 1.20)	
Living with partners	299	1.02 (0.88, 1.18)	
**Education level**			0.622
Less than 9th grade	323	1.05 (0.95, 1.16)	
High school graduate	1490	1.11 (1.05, 1.17)	
College graduate/ above	2664	1.11 (1.05, 1.16)	
**Family PIR**			0.103
0 - 1.18	1329	1.08 (1.02, 1.13)	
1.19 - 2.82	1332	1.15 (1.08, 1.22)	
2.83 - 5	1334	1.01 (0.88, 1.15)	
**Health insurance**			0.512
no	884	1.13 (1.06, 1.21)	
yes	3587	1.10 (1.06, 1.14)	
**Diabetes mellitus**			0.022
no	3859	1.08 (1.04, 1.12)	
yes	427	1.21 (1.10, 1.32)	
**Smoking status**			0.483
Never smoking	2910	1.11 (1.05, 1.17)	
Current smoking	759	1.06 (1.00, 1.13)	
Former smoking	803	1.12 (1.05, 1.20)	
**Alcohol consumer**			0.277
no	1680	1.08 (1.01, 1.15)	
yes	2791	1.12 (1.08, 1.17)	
**Hypertension**			0.291
no	2764	1.12 (1.06, 1.17)	
yes	1709	1.07 (1.02, 1.13)	
**Hyperlipemia**			0.724
no	2799	1.11 (1.06, 1.16)	
yes	1656	1.09 (1.03, 1.16)	

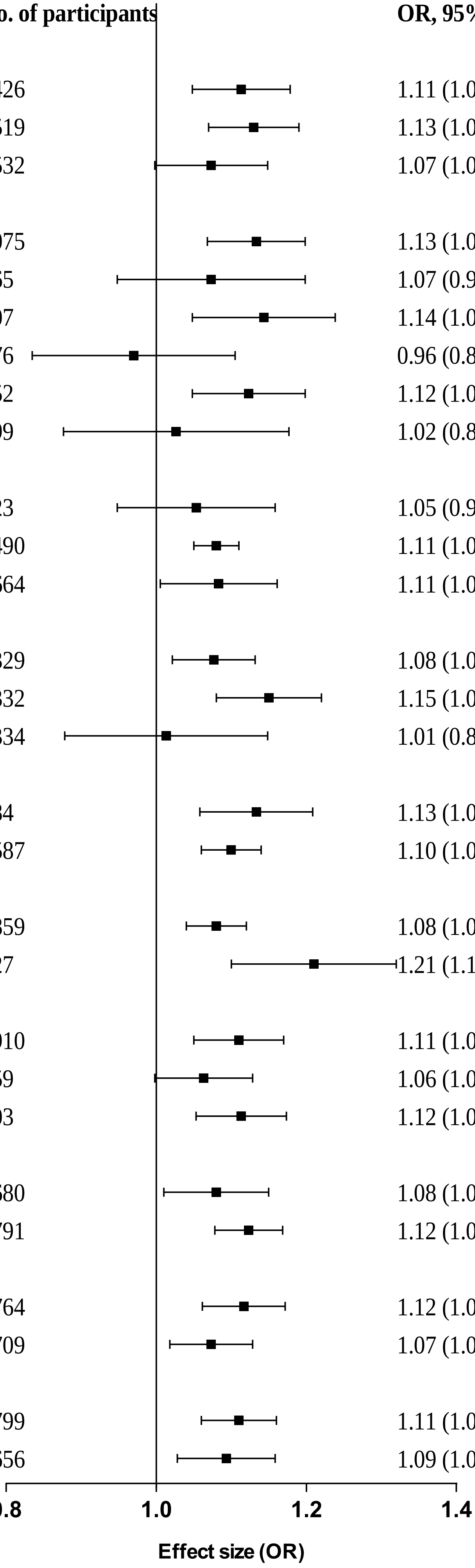

(S4)

Characteristics	No. of participants	OR, 95%CI	*P* for interaction
**Age (year)**			0.400
20 - 39	1426	1.14 (1.02, 1.27)	
40 - 58	1519	1.06 (1.00, 1.13)	
59 - 80	1532	1.02 (0.92, 1.15)	
**Marital status**			--
Married	2075	1.07 (0.96, 1.18)	
Widowed	465	1.03 (0.89, 1.18)	
Divorced	607	1.06 (0.95, 1.17)	
Separated	176	1.09 (0.92, 1.29)	
Never married	852	1.07 (0.95, 1.20)	
Living with partners	299	1.23 (1.03, 1.47)	
**Education level**			0.650
Less than 9th grade	323	1.10 (0.95, 1.26)	
High school graduate	1490	1.04 (0.96, 1.13)	
College graduate/ above	2664	1.10 (1.02, 1.18)	
**Family PIR**			0.357
0 - 1.18	1329	1.04 (0.97, 1.12)	
1.19 - 2.82	1332	1.07 (0.97, 1.17)	
2.83 - 5	1334	1.18 (1.01, 1.37)	
**Health insurance**			0.835
no	884	1.07 (0.96, 1.19)	
yes	3587	1.09 (1.03, 1.15)	
**Diabetes mellitus**			0.997
no	3859	1.09 (1.03, 1.15)	
yes	427	1.09 (0.95, 1.25)	
**Smoking status**			0.868
Never smoking	2910	1.07 (0.99, 1.15)	
Current smoking	759	1.09 (1.01, 1.18)	
Former smoking	803	1.05 (0.93, 1.18)	
**Alcohol consumer**			0.631
no	1680	1.10 (1.02, 1.20)	
yes	2791	1.08 (1.01, 1.15)	
**Hypertension**			0.083
no	2764	1.12 (1.04, 1.20)	
yes	1709	1.02 (0.95, 1.09)	
**Hyperlipemia**			0.266
no	2799	1.10 (1.03, 1.18)	
yes	1656	1.04 (0.97, 1.13)	

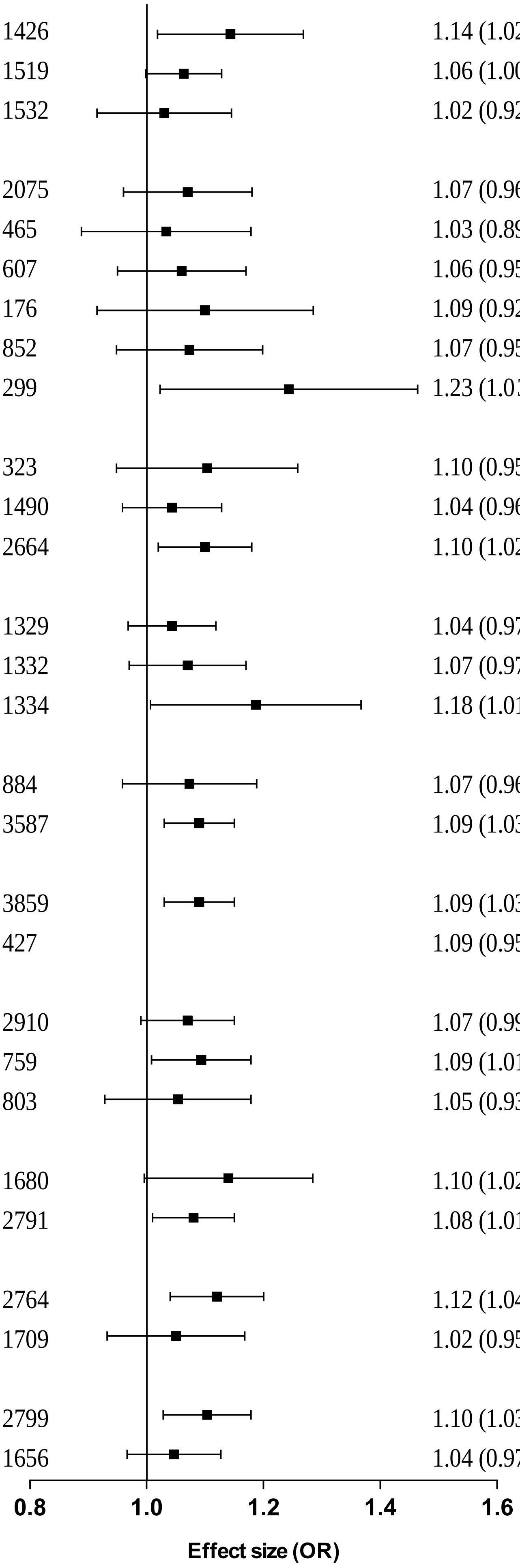

Supplement: Supplementary 2 — Figure S1: forest plot showing the odds ratios, confidence intervals, and p value for the interaction of SAD and depression in the different groups. Figure S2: forest plot showing the odds ratios, confidence intervals, and p value for the interaction of SAD and moderate depression in the different groups. Figure S3: forest plot showing the odds ratios, confidence intervals, and p value for the interaction of SAD and moderate severe depression in the different groups. Figure S4: forest plot showing the odds ratios, confidence intervals, and p value for the interaction of SAD and severe depression in the different groups. [file 9624106.f2.pdf]
